# The Use of Speculative Fiction in Future-Focused Health Care Research

**DOI:** 10.2196/89699

**Published:** 2026-05-22

**Authors:** Sjors Groeneveld, Jobke Wentzel, Melissa Laurens, Lisette van Gemert-Pijnen, Rudolf M Verdaasdonk, Harmieke van Os-Medendorp, Marjolein den Ouden

**Affiliations:** 1 Research Group Technology, Health & Care, School of Social Work, Saxion University of Applied Sciences, Postbox 70.000, Enschede, Overijssel, 7500 KB, The Netherlands, 31 880198888; 2 TechMed Center, Health Technology Implementation, University of Twente, Enschede, The Netherlands; 3 Research Group ICT Innovation in Care, Windesheim University of Applied Sciences, Zwolle, The Netherlands; 4 Research Group Care and Technology, Regional Community College of Twente, Hengelo, The Netherlands; 5 Centre for eHealth and Wellbeing Research, Section of Psychology, Health and Technology, University of Twente, Enschede, The Netherlands; 6 Faculty Health, Sports, and Social Work, Inholland University of Applied Sciences, Amsterdam, The Netherlands; 7 Spaarne Gasthuis Academy, Haarlem, The Netherlands

**Keywords:** health care research, speculative fiction, Futures Studies, research tools, narratives, health technology, digital health

## Abstract

Health care is undergoing rapid change due to digitalization, artificial intelligence, data-driven decision-making, and shifting patient needs. These developments raise complex ethical, social, and organizational questions that cannot always be addressed by conventional research methods alone. There is a growing need for tools that help stakeholders imagine alternative futures to surface underlying values. Futures studies and speculative fiction respond to this need by presenting “what if” scenarios that make uncertain futures concrete and discussable, enabling dialogue among diverse stakeholders such as health care professionals, researchers, policy makers, and decision makers. This paper examines how speculative fiction can be used as a structured yet imaginative tool in health care research. It positions speculative fiction as a tool within futures studies and participatory research approaches, outlining its conceptual grounding and clarifying its role that stimulates interpretation and reflection within methods such as focus groups, workshops, and surveys. In this way, speculative fiction complements established approaches such as human-centered design, contextual inquiry, and value specification by addressing what does not yet exist and making abstract future issues tangible. The paper presents a case study, The Digital Data Divide, in which speculative fiction was used to explore the use of personal data in health care. Two contrasting short films were developed to stimulate dialogue and invite participants to reflect on associated values. Insights from this case are structured into a 6-step practical reporting guide: determining whether speculative fiction fits the research aim, choosing an appropriate form of speculative fiction, creating or selecting speculative scenarios, engaging participants, analyzing responses, and sharing and disseminating results. Across these steps, the paper discusses methodological and ethical considerations, including alignment between scenarios and study aims, balancing utopian and dystopian elements, questions of plausibility and interpretability, and the need for researcher reflexivity. Overall, this paper contributes to the growing discussion on future-oriented tools in health care research by showing how speculative fiction can help address complex and uncertain challenges in ways that are accessible to a wide range of stakeholders and that support dialogue across perspectives, provided that its use is ethically transparent, methodologically explicit, and carefully reported. The paper concludes with a call to other researchers to also experiment with speculative fiction and to share their experiences with the health care research community to learn and advance its use.

## Introduction

Health care is changing fast because of new technologies, social developments, and changing patient needs [1]. Digitalization, artificial intelligence (AI), data-driven decision-making, and new models of care delivery are reshaping how health care is organized and how professionals work [2]. At the same time, aging populations, rising multimorbidity, workforce shortages, and increasing expectations for personalized care add further pressure to the system. These developments generate complex questions that are not purely technical but also ethical, social, and organizational. For example: How should personal data be used responsibly? What forms of professional judgment will matter most in data-driven health care? How can care remain person-centered when technologies become more dominant? And how should changing roles and responsibilities be negotiated between professionals, patients, and digital systems? Many of these questions cannot be fully addressed through conventional research alone and benefit from approaches that present alternative futures, challenge underlying assumptions, and help define ethical boundaries by engaging stakeholders in collective sense-making around uncertain futures [3-5]. This is particularly important for ethical or value-based issues, where the aim is not to predict the most *probable* future but to explore and discuss (multiple) *possible* futures in a structured, open, and reflective manner [5,6]. Futures studies and speculative fiction can help do this.

The aim of this paper is to provide inspiration and guidance on how speculative fiction can be used in health care research. Specifically, this Viewpoint (1) positions speculative fiction as a structured probe within futures-oriented health research and (2) offers a practical, stepwise guide illustrated through the case of The Digital Data Divide. By doing so, we clarify both the conceptual rationale and the practical application of speculative fiction for health care researchers seeking to engage stakeholders in reflection on possible futures.

## Speculative Fiction: A Tool for Exploring Possible Futures

*It is 2055. Chronic diseases like diabetes and heart failure are managed entirely at home. AI monitors vitals in real time, robots assist with daily care, and virtual nurses check in via hologram. Hospitals focus only on emergencies, while neighborhood health hubs handle the rest.*

For some, this future promises efficiency and independence; for others, it raises urgent concerns: what happens to human connection in care? Who is accountable when algorithms fail? How do we ensure that no one gets left behind? This scenario isn’t a prediction; it is speculative fiction, a broad term for stories, images, or experiences that deliberately depart from consensus reality to explore alternative futures and their consequences [7]. Unlike traditional forecasting that projects the future based on the most *probable* developments and quantitative trends, speculative fiction aims to explore *possible* futures and its consequences [8]. This distinction resonates with established futures studies frameworks, such as Voros’ Futures Cone [9]. Speculative fiction uses imagination to reflect on questions, from ethics to equity, and to spark conversations about the world we want...or want to avoid [10].

We recognize that speculative fiction overlaps with adjacent traditions such as speculative and critical design, design fiction, and experiential futures [11-14]. While these approaches differ in disciplinary roots and primary outputs, they share a commitment to engaging audiences through encounters with imagined futures. In this paper, we use speculative fiction as an umbrella term for narrative, visual, experiential, and material forms when they function primarily as stories about a possible future world. Our focus is therefore less on design as production and more on fiction as a narrative device that enables reflection, collective sense-making, and dialogue within health care research.

Speculative fiction is widely used beyond academic research. It thrives in culture and society. From dystopian television series such as “Star Trek,” “Black Mirror,” or “The Handmaid’s Tale” to computer games and visionary works of science fiction literature, it captivates audiences by exploring alternative realities and posing provocative “what if” questions. These stories challenge our assumptions about technology, ethics, and society, often serving as a mirror to reflect on contemporary anxieties or aspirations. There is a body of educational and sociological scholarship that suggests that fictional narratives, such as science fiction [15,16], can function as powerful sense-making tools. By inviting readers and viewers to temporarily inhabit alternative perspectives and social worlds, these stories render abstract or complex societal dynamics more tangible and emotionally resonant. Their capacity to create cognitive and moral distance, while simultaneously fostering identification and critical reflection, may help explain why speculative fiction is particularly well suited to exploring possible futures of technology and care.

For decades, Futures Studies have systematically explored possible, probable, and (un)desirable futures to inform policy, strategy, and societal development [17,18]. In this paper, we position speculative fiction as a tool within this established academic field of Futures Studies. Speculative fiction as a tool is not a data collection method: it acts as a probe that stimulates interpretation and reflection within methods of data collection such as focus groups, workshops, facilitated dialogues, or surveys. Speculative fiction thereby complements existing approaches such as participatory methods, contextual inquiry, value specification, and design thinking. While these approaches share the goal of uncovering values, interests, and perspectives [19,20], they typically focus on current realities or probable futures. Speculative fiction, however, excels in addressing what does not yet exist. For example, contextual inquiry seeks to understand current needs and behaviors, but speculative fiction pushes this exploration into hypothetical futures. Similarly, value specification and service road mapping aim to clarify priorities and map pathways, but speculative fiction introduces unfamiliar or disruptive contexts, forcing stakeholders to confront uncertainties and ethical dilemmas that otherwise might be overlooked. Even within design thinking’s iterative cycles, speculative approaches act as a “what if” sandbox [12], enabling teams to stress-test ideas against possible future conditions. In this way, it complements these approaches by making the intangible tangible.

By deploying speculative fiction in health care research, we align with the field’s core goal of fostering democratic participation and cross-sectoral dialogue [3], while cultivating futures literacy: the competence to reflect on diverse attitudes toward the future and generate alternative scenarios to navigate complexity and uncertainty [17,21].

## The Role of Speculative Fiction in Health Care

As discussed earlier, speculative fiction can complement established research methods by addressing what does not yet exist. An example from health care is the CeHReS Roadmap, a health care–specific, design-oriented approach that emphasizes contextual values and stakeholder needs early in the development of eHealth technologies [22]. While the CeHRes Roadmap excels in mapping current priorities and behaviors, it focuses on existing realities or imaginable situations. Speculative fiction adds a critical layer by projecting these values and needs into hypothetical futures. For example, while the CeHRes Roadmap might uncover a care team’s current frustrations with digital tools, speculative fiction can explore how those frustrations might evolve in a future where AI-driven diagnostics or robotic care assistants fundamentally reshape their roles. By creating speculative scenarios, it enables stakeholders, including those who rarely engage with future-oriented questions in their daily work, such as frontline caregivers or administrative staff, to confront complex dilemmas, uncover hidden assumptions, and co-design adaptive solutions. Speculative fiction’s strength lies in its ability to democratize futures thinking, surface unspoken concerns, and bridge abstract trends and action. Speculative fiction takes many forms, each offering unique ways to engage with possible futures and their implications. For example, initiatives such as the “Expo Future of work in health care and wellbeing,” developed by a Dutch center of expertise for long-term care, used an interactive, traveling exhibition to engage health care professionals in exploring future scenarios for the care sector [23]. The expo presents 4 speculative visions of care work in 2040, combining visual storytelling, interactive installations, and discussion prompts to start dialogue about technological, organizational, and societal changes. Although inspiring, such projects are still an exception rather than the rule.

While its potential is vast, a recent scoping literature review [3] shows that Futures Studies primarily focus on macro-level themes (eg, national strategies, pandemic preparedness, and workforce dynamics). These studies highlight *what* needs to be explored but often overlook *how* tools such as speculative fiction can be deployed within organizations to mobilize internal stakeholders in cocreating their futures. Building on the established value of speculative fiction, the following section of this paper shifts from theory to application, offering practical insights learned from a case study.

## Practical Insights Learned in Using Speculative Fiction

### Overview

This section focuses on the practical aspects of using speculative fiction in research. This is illustrated through the introduction of a case project, The Digital Data Divide, from which practical insights are drawn and structured into six steps: (1) determining whether speculative fiction aligns with the research aim, (2) choosing the form of speculative fiction, (3) creating or selecting speculative fiction scenarios, (4) engaging participants during data collection, (5) analyzing responses, and (6) sharing results. These steps are subsequently synthesized into a 6-step reporting guide to facilitate clarity, transparency, and transferability in future applications.

### Case: The Digital Data Divide

The growing ability to collect and analyze personal data is transforming many sectors, including health care [24]. Data from wearable devices, smart home sensors, and digital health applications increasingly inform clinical decisions and public health strategies [25]. While these developments hold promise for more personalized and efficient care, they also raise complex questions about privacy, autonomy, trust, and the ownership of data [26]. These issues are not only technical but also social and ethical, requiring spaces for dialogue and reflection across different parts of society.

**Figure 1.**
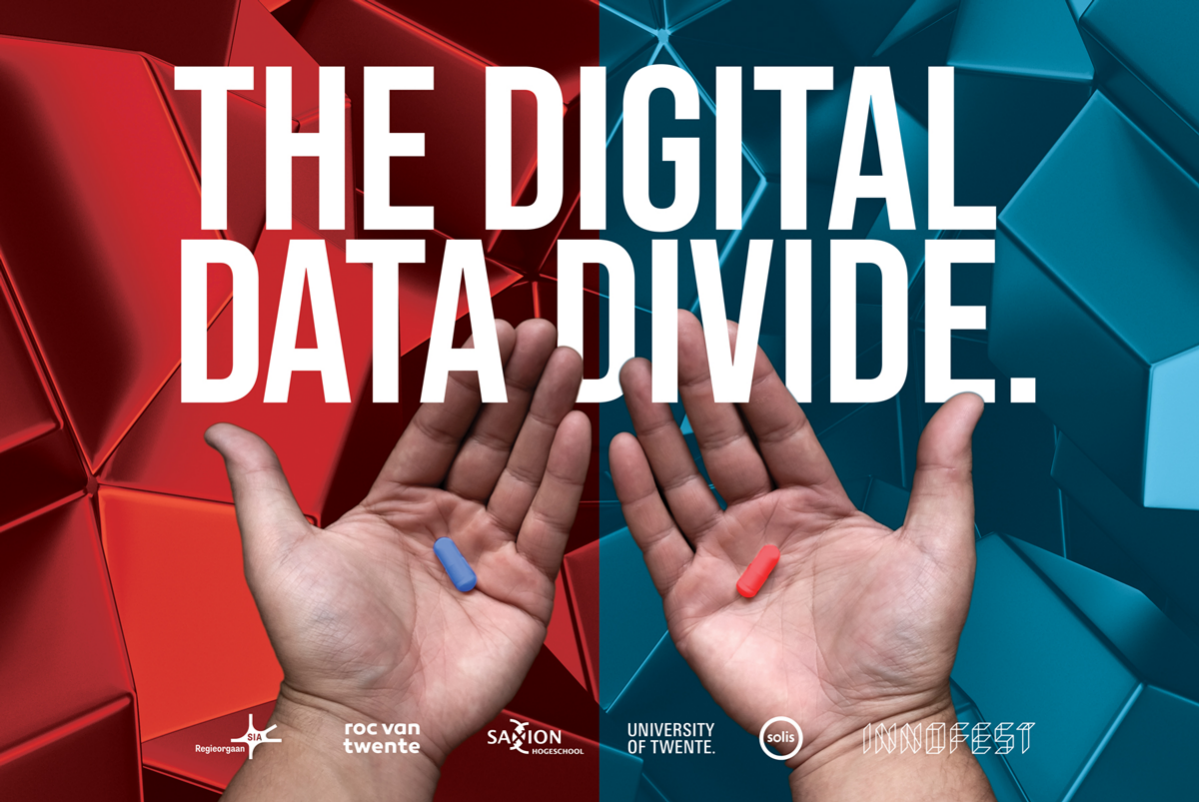
The Digital Data Divide poster.

To create such a space and engage a broad audience in discussions about data-driven futures, the project The Digital Data Divide is developed [27] (see Figure 1 for the project poster). This project is designed as an interactive speculative experience that can be used in both public festival contexts and more structured educational or research settings, using speculative fiction to provoke reflection and dialogue. Participants begin the experience as they are invited to make a symbolic choice between a red and a blue pill, each representing a different future scenario. Based on their choice, participants view 1 of 2 short speculative films that follow an ordinary person (Maarten or Maartje) throughout a day in a data-saturated society. In both films, personal data play a central role in everyday life. However, in the “red” scenario, data systems subtly work against the person, shaping opportunities, reinforcing inequalities, enabling surveillance, and limiting autonomy and agency over personal data. In the “blue” scenario, data infrastructures are depicted as supportive, seemingly enhancing well-being, safeguarding privacy, and enabling choice and control. Themes such as privacy, autonomy, surveillance, commercial data use, and social (in)equality recur in both films but are framed in contrasting ways. Differences in aesthetic elements such as color palette and music further reinforce the emotional tone of each world.

After viewing the films, participants reunite in a shared space and use dialogue cards to discuss what they had seen; reflect on the ethical, personal, and societal implications of these imagined futures; and make underlying values, assumptions, and emotional responses explicit. The contrasting scenarios surfaced participants’ underlying values and assumptions about technology and data, prompting reflection not only on desirable futures but also on the societal conditions required to achieve them.

**Figure 2.**
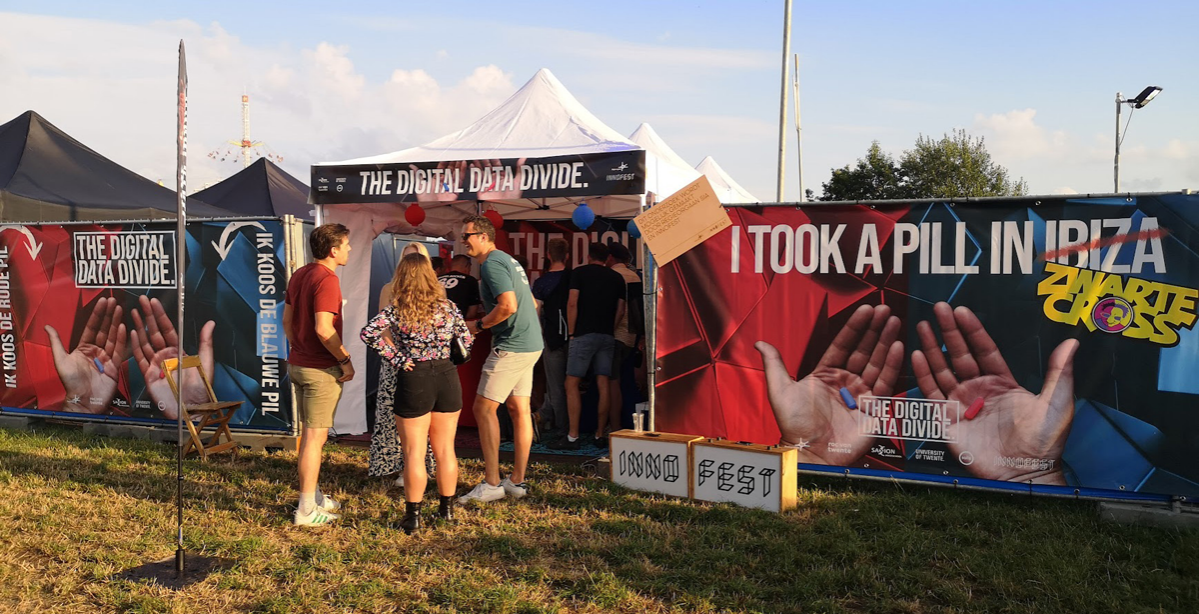
The Digital Data Divide at a festival setting.

The project is used across a wide variety of contexts, including public festivals, educational institutions, research projects, and professional events. Figure 2 illustrates its appearance in a festival context. Over the course of time, The Digital Data Divide reached a diverse audience, from health care professionals and students to members of the public. By situating the conversation in both informal public settings and more structured educational or research contexts, the project aims to lower the barrier for participation, while also enabling deeper reflection. Across settings, it supports participants in exploring and articulating views beyond their usual professional or social boundaries.

Across its implementations, The Digital Data Divide has been used in 2 distinct but related contexts. In public festival settings, the project functioned as an exploratory live experience to test whether speculative fiction could stimulate dialogue in informal environments. The project reached members of the general public from diverse backgrounds. Participants provided feedback through brief flyers containing visual analogue scales (eg, perceived awareness of the impact of data and evaluation of the experience) and open comment fields. Responses were collected on-site and qualitatively clustered to identify recurring reflections and suggestions for improvement.

In a later, more structured qualitative focus group study with international health care students (n=40), the same films were used as prompts for moderated discussion. These sessions were recorded and analyzed using an inductive thematic approach. While the festival implementation primarily served exploratory development purposes, the focus group setting allowed for deeper analysis of how participants reasoned about imagined data-driven futures.

Across both applications, several recurring tensions became visible. Participants valued the use of contrasting extremes, noting that the utopian or dystopian framing helped clarify their own positions. The films heightened awareness of the societal impact of personal data, though responses ranged from concern to skepticism about plausibility. Some participants experienced the format as highly thought-provoking, while others expressed a desire for clearer facilitation or more practical information about current realities. These insights informed further refinement of the dialogue format. In particular, we became more attentive to the delicate balance between realism and imaginative projection: scenarios need to feel credible enough to enable identification, while remaining sufficiently speculative to open space for critical reflection. This realization shaped our recommendation to carefully calibrate plausibility and contrast when using speculative fiction as a structured tool for future-oriented dialogue.

### Step 1: Determine Whether Speculative Fiction Fits the Research Aim

The first step in using speculative fiction is to establish whether the research question benefits from imaginative exploration. Speculative approaches work particularly well for topics marked by uncertainty, ethical complexity, or potential changes in (professional) practice, especially when questions involve ambiguity, value tensions, and several plausible outcomes [28,29]. In health care, this could include (the use of) emerging technologies, evolving models of patient care, or shifts in professional roles, responsibilities, and ethical boundaries. Rather than predicting the most likely future, speculative fiction invites participants to imagine a possible future and reflect on its desirability, risk, and implications, resulting in rich insights into the underlying values and preferences that shape how people relate to future technologies. Speculative fiction is less suitable for highly defined operational questions or established interventions. A useful guiding question is: Does this topic involve futures for which no empirical data yet exist, but where societal and ethical reflection is necessary?

In The Digital Data Divide project, the research focus centered on the increasing use of personal data in health care and its ethical and societal consequences. Other possible topics could be the future role of nurses in AI-supported care, scenarios for end-of-life decision-making, or future models of empathy and access to care. Establishing a focus at this conceptual level ensures that speculative fiction is not used as a storytelling exercise alone but as a structured method for exploring emerging health futures.

### Step 2: Choosing the Form of Speculative Fiction

After identifying the research focus, the next step is to select an appropriate form. Speculative work can take many shapes including written narratives, audiovisual media, interactive formats, and design fiction artifacts [30]. Although these forms vary widely, we propose grouping them into 4 overarching categories including anticipated strengths and limitations to support practical comparison (Table 1). Each form supports a different kind of audience, mode of participation, and depth of discussion, and requires different resources. Through written formats (ie, short stories, diary fragments, and poetry), it delves into deep narrative worlds, using speculative storytelling to question assumptions, reimagine systems, and propose bold alternatives [31]. Written narratives often work well for individual reflection and deeper introspection, while audiovisual media such as short films or animations can evoke strong emotional responses and intellectual engagement that provide a powerful starting point for shared dialogue [32]. Interactive or performative formats, for example, installations or live role-playing through participatory theater, are particularly effective for larger or public audiences where collective participation and experiential learning are desired. Design fiction artifacts concretize future scenarios by introducing tangible objects or representations, enabling participants to engage in concrete discussions about aspects such as usability and everyday practice.

When deciding which form to use, we suggest considering four criteria: (1) the audience (individual, small groups, or larger groups), (2) the desired mode of participation (reflection, group dialogue, or public engagement), (3) the practical feasibility and resources available, and (4) the desired depth of discussion or analysis.

**Table 1.** Examples of possible forms of speculative fiction.

Form of speculative fiction	Most suitable for	Strengths	Considerations or limitations
*Written narratives* (short stories, diary fragments, and letters from the future)	Individual reflection; sensitive or value-laden topics	Supports introspection and slower, thoughtful engagement	Requires reading time and capacities; less suitable for large or public audiences
*Audiovisual media* (short films, animations, and illustrated scenarios)	Group dialogue, emotional engagement, and mixed audiences	High accessibility, strong emotional impact, and effective for shared reflection	Resource-intensive, requires production skills, and risk of oversimplification
*Interactive formats* (installations, role-playing, experiential futures, and theater play)	Large groups, public events, and festivals	Immersive and memorable; encourages active participation	Logistical complexity, requires facilitation, and harder to standardize
*Design fiction artifacts* (prototypes, speculative objects, interfaces, and fictional brochures)	Stakeholder workshops, design reviews, policy dialogue, and education	Makes future technologies feel concrete, helps discuss assumptions about usability, and everyday practice	Risk of being interpreted as “the” solution, requires careful framing, and production effort varies depending on fidelity

In The Digital Data Divide project, original film-based scenarios were used to create an emotionally engaging experience suitable for both informal public festivals and more educational or research environments. The choice for film supported accessibility, narrative depth, and emotional relatedness. Two contrasting short films (1 utopian and 1 dystopian) were made with the goal to start a dialogue on relevant values from multiple perspectives. This contrast helped in the discussion to reflect on what is important for you as a person in a *possible* future, instead of finding consensus as participants in the most *probable* future.

### Step 3: Creating or Selecting Speculative Fiction Scenarios

Once the form is chosen, the next step is deciding whether to develop new speculative material or adapt existing fictional work. Creating original speculative narratives offers flexibility and ensures alignment with the research aims, but it requires more time, resources, and often interdisciplinary collaboration. When taking this route, the competency of the creators becomes crucial: scenario development benefits from a combination of domain knowledge (such as health care, ethics, laws, or regulations), creative skills (such as storytelling and design), and methodological awareness to ensure that the material is relevant, balanced, and responsibly framed. Original speculative scenarios can be created individually or in cocreation with a wide range of stakeholders, including not only domain experts and end users but also designers, ethicists, creatives, and other voices who can meaningfully shape how a future is imagined. Individual writing allows for very specific controlled narratives, while group cocreation supports diversity of perspectives and greater creative range. Creative workshops, often including structured ideation methods such as brain writing, futures wheel, SCAMPER, or the Experiential Futures Ladder [14,33], can help translate abstract concerns into concrete situations, characters, and moral dilemmas.

Using existing stories or cultural references can reduce development time, but it also requires careful assessment of the material’s quality and suitability, a concern that aligns with methodological discussions in vignette studies [34]. Researchers should examine whether the fiction aligns with the research focus, offers sufficient nuance for meaningful reflection, and presents a balanced mix of positive and negative elements. It is also important to consider whether the material avoids unnecessary bias and does not unintentionally steer participants toward a particular viewpoint. Although concepts such as validity and reliability take on a different form when creating or selecting speculative fiction scenarios, focusing on plausibility and interpretability rather than factual accuracy, transparency about narrative choices, and ethical intent remains crucial to ensure that (adapted) material is used in a responsible and balanced way. In this regard, involving intended participants or end users during scenario development or selection can help ensure that scenarios feel realistic, balanced, and meaningful for those who will later respond to it.

In The Digital Data Divide project, speculative scenarios were developed through cocreation in a multidisciplinary session in which researchers, health care professionals, ethicists, students, designers, and film professionals explored emerging questions around personal data in health care. This informal yet structured session generated early narrative ideas, ethical dilemmas, and possible character storylines. A scriptwriter then translated these insights into draft scripts. Filmmakers, actors, and a composer were involved to refine tone, pacing, emotional resonance, and visual language. Importantly, the development process moved iteratively from concept to scenario to finished film. This shows that speculative scenarios are not static elements but coconstructed narratives formed by a wide range of involved people, creative expertise, and production realities.

### Step 4: Engaging Participants During Data Collection

Engaging participants is essential for turning speculative scenarios into meaningful insights rather than leaving them as stand-alone stories [12,14,29]. To ensure that speculative scenarios contribute to the research aim, participants need structured support to move from simply reading, viewing, or participating in a fictional future to considering what it might mean for their own practice, values, or professional identity. Clear prompts, guiding questions, or dialogue tools help make this transition explicit. Speculative fiction is most effective when participants are encouraged not only to react to a story but also to position themselves within the imagined situation and articulate which futures they consider desirable, problematic, or uncertain. In this way, the speculative element becomes a tool for research-relevant reflection and meaning-making. Ethical considerations are essential as speculative scenarios can influence how participants think or feel about an issue, especially when they include strong dystopian or utopian elements. Clear informed consent [35], openness about the purpose of the scenario, and attention to potential emotional impact help ensure that speculative materials are used responsibly.

The Digital Data Divide project was deliberately designed to be accessible and engaging, suitable for both diverse public audiences and more structured research or educational environments. In both applications, participants first viewed contrasting speculative films individually, after which structured reflection was facilitated in a shared setting. Dialogue cards encouraged participants to express emotional responses, consider ethical tensions, and explore how data-driven futures might influence their professional roles or personal autonomy. This process deepened the conversation and linked the speculative narrative to the ongoing construction of professional identity and practice.

In the festival setting, feedback was collected using brief hard copy flyers with visual analogue scales and open comment fields. This pragmatic method was intentionally selected to suit an informal festival context, where lengthy questionnaires or structured interviews would have created participation barriers. In the qualitative focus group study, the same films were used as prompts for moderated discussion, and conversations were recorded and analyzed qualitatively. Together, these applications illustrate how speculative scenarios can function flexibly as a tool across different contexts.

Given that speculative scenarios may evoke sensitive reflections, ethical considerations were addressed in both applications of The Digital Data Divide, although in different ways reflecting their respective contexts. As said, the festival setting functioned as an exploratory live experience rather than a formal research study. No identifiable personal data were collected, and no audio or video recordings were made. Participants were informed in advance (both verbally and through the flyer) about the nature of the experience, including the presentation of contrasting future scenarios and the voluntary character of participation. Feedback was provided using the brief hard copy flyers, and the anonymous responses were stored on a protected research drive. Prior to the festival experience, the approach was discussed with ethics and privacy advisors to consider potential personal (data) risks. For the focus group study (where the films were used as one component within a broader qualitative research design), formal ethical approval was obtained through the relevant institutional review procedures at participating universities. Participants received an information letter and signed informed consent prior to participation. They were explicitly informed about the recording of the sessions, their right to withdraw at any time without consequences, and the handling of their data. Recordings were encrypted, transcribed without identifying information, and stored on a secure, password-protected research drive accessible only to the research team.

### Step 5: Analyzing Responses

The fifth step concerns the analysis of participant responses. As noted earlier, speculative fiction is not a data collection method on its own; it is a tool that functions as a probe that triggers interpretation and reflection within methods of data collection such as focus groups, workshops, facilitated dialogues, or surveys. The data analysis of discussions based on speculative fiction may draw on approaches such as thematic analysis [36], narrative analysis [37], or mixed methods designs [38]. The focus is typically on meaning-making: how participants articulate values, ethical concerns, and desirable or undesirable futures. Rather than searching for “correct” answers, analysis aims to surface patterns in reasoning, identity formation, and expectations about emerging technologies. In mixed methods approaches, these qualitative insights can be combined with quantitative measures (such as surveys or rating scales) to provide a more comprehensive understanding of how participants make sense of the fictional futures.

The exploratory festival implementation of The Digital Data Divide necessarily introduced limitations. Measures of awareness were simplified, context factors (eg, festival atmosphere and time of day) that could influence responses, and the open nature of the setting could introduce information bias. These constraints raise important questions about validity and reliability in open, participatory contexts. Nonetheless, the method demonstrated that speculative fiction could generate meaningful insights outside traditional research environments, particularly when the goal is exploratory sense-making rather than precise causal inference. In the focus group study where the films from The Digital Data Divide project were used with students, the resulting qualitative data were analyzed using an inductive thematic approach, through which themes were constructed from the students’ reflections. While the analysis followed a conventional qualitative procedure, the goal remained exploratory: to understand how participants reasoned about imagined futures, rather than explanatory to treat their responses as “data” about current reality.

### Step 6: Sharing Results

A final consideration is how to share insights generated through speculative fiction. Unlike some traditional research, speculative approaches do not only inform scholarly debate but also aim to circulate ideas, provoke reflection, and shape broader conversations about the future in society. Dissemination therefore extends beyond academic publications to include public exhibitions, community dialogue sessions, educational activities, and creative media outputs: formats that in some cases resemble those used in practice-based research. Returning insights to society is particularly relevant when imagined futures may influence real ones: presenting speculative scenarios and the reflections they generate can contribute to collective sense-making and democratic discussion about emerging technologies. In this context, collective sense-making refers to the shared interpretive process by which groups negotiate meanings, articulate underlying values, and coconstruct understandings of plausible futures. Drawing on literature on sense-making, the collective aspect emphasizes how participants collaboratively make sense of imagined futures rather than merely expressing isolated opinions [39]. By encountering contrasting scenarios and discussing them together, participants do not only reflect individually but also shape shared interpretations and concerns, supporting broader insights about the impact of technology.

In The Digital Data Divide project, this principle guided the choice to present findings not only in academic settings but also through continued public engagement at festivals, health care organizations, and educational programs. By sharing results in the same open spaces where the dialogue began, the project aimed to extend reflection, spark new conversations, and reinforce a feedback loop in which speculative visions and societal responses mutually shape one another.

### Binding It Together

Together, the aforementioned insights, structured into 6 steps, show an example of how speculative fiction can be used as a structured but creative research tool in health care. By choosing a future-focused question, selecting the right speculative format, creating or selecting scenarios, involving participants, analyzing their reflections, and sharing results with society, speculative fiction goes beyond storytelling and becomes a tool for future-focused research. It builds ethical awareness, critical thinking, and open discussion in areas where evidence is limited and the future is uncertain. Unlike traditional scenario approaches, which often aim to map trends or outline plausible developments, speculative fiction creates experiential and value-rich situations that help participants feel and interpret possible futures. To enhance transparency and reusability, Table 2 provides a compact reporting guide aligned with the 6 steps. Researchers using speculative fiction may consider reporting on the following elements. This checklist is not intended as a rigid protocol but as a guide to support transparent and context-sensitive reporting when speculative fiction is used as a research tool.

**Table 2.** Six-step reporting guide for using speculative fiction in health care research.

Step	What to report	Key considerations
1. Research aim	Why speculative fiction was chosen and its foreseen contribution	Nature of uncertainty, ethical/value tensions, and intended contribution (exploratory, dialogical, and analytical)
2. Form of speculative fiction	Chosen format and rationale	Intended audience, level of participation, resources, and depth of engagement
3. Scenario development	How scenarios were created or selected	Cocreation process, balance of perspectives, plausibility checks, mitigation of bias, and involvement of stakeholders
4. Participant engagement	Facilitation approach and data collection	Prompts or dialogue tools used, setting of data collection, informed consent procedure, emotional safeguards, ethical issues, and handling the data
5. Analysis approach	How responses were interpreted	Qualitative or quantitative methods, analytic lens (eg, thematic and narrative), and reflexivity of researchers
6. Sharing and dissemination	How insights were circulated	Academic outputs, public engagement, feedback loops, and contribution to collective sense-making

## Discussion

### Principal Findings

This paper demonstrates how speculative fiction can support the exploration of uncertain or sensitive future issues in health care, aligning with the increasing need for future-oriented approaches to address the sector’s wicked problems [22,40]. Many intended participants, such as patients, informal caregivers, and health care professionals, may find it difficult to envision future scenarios or anticipate their consequences. Because these groups are often most affected by change, methods are needed that help unlock their perspectives and latent knowledge and enable creative, reflective contributions [41]. Speculative fiction bridges this gap by offering concrete, imaginative future scenarios that people can react to, rather than asking them to invent those futures themselves. This creates a more inclusive starting point, giving participants (regardless of education, experience, or role) an equal seat at the table. By making hypothetical futures tangible, speculative fiction supports those who struggle to express abstract ideas or anticipate change, enabling richer discussions about values, concerns, and desirable directions for health care [42]. Speculative fiction thereby complements widely used participatory and design-based methods such as Human-Centered Design, which rely on participants’ articulating needs and imagining alternatives [43,44].

In addition, much participatory research relies on very verbalized methods: participants need to be able to comprehend the language the researcher uses and need to be able to express their own thoughts, desires, values, and needs in words. Not all participants can do this with ease, leading to possible biases [40]. Researchers have sought to overcome such biases by using visual stimuli to provoke thoughts and encourage idea-sharing among their participants using visual methods such as photo elicitation [45], which demonstrate how images can help participants express experiences and perspectives that are difficult to verbalize through conventional interviews alone. Speculative fiction can complement frameworks such as Design Thinking [46] by introducing future scenarios when imagining or verbalizing such situations often lies beyond the capacity of participants. In this sense, speculative fiction differs from other tools by presenting a possible future for participants to respond to, rather than asking them to construct it themselves. Speculative fiction enhances inclusivity by providing multiple modes of sense-making (verbal, visual, narrative, and experiential). This allows participants to engage with future scenarios without relying solely on abstract reasoning or verbal articulation. This enables involvement and opinion-forming among groups who may be difficult to engage through traditional research methods. Speculative fiction thus creates a shared entry point for exploring future directions across system levels. At the micro level, it helps individuals reflect on their values, identity, and responses to future situations. At the meso level, it enables teams and organizations to build shared visions and explore strategic choices with internal stakeholders. At the macro level, it can inform policy debates and spark wider societal dialogue [47]. In this way, speculative fiction supports meaningful conversations about the future across all levels of the health care system.

In their scoping review, Meskó and colleagues [3] conclude that a more systematic or guideline-based approach is needed for the practice of future methods. We wish to elaborate on this call, based on our experiences, with speculative fiction. *First*, in line with good and ethical research practice, transparency about the researcher’s goal and chosen approach is key [35]. Speculative fiction is a powerful tool: it can trigger strong emotional responses, shape opinions, and foreground certain futures while obscuring others. Similar debates in bioethics highlight that speculative work can serve different purposes, and that standards for impact and good practice should be adjusted accordingly [48]. Researchers’ own values and assumptions inevitably influence the scenarios they create or select, as well as how participants respond to them. Much like in qualitative research, this calls for continuous reflection on the own subjective role of researchers [49]. Involving multiple viewpoints, iterating between data and interpretation, and using triangulation can help safeguard balance and transparency. We argue that research papers that use speculative fiction should explicitly incorporate these ethical considerations, as this will help develop responsible, transparent, and effective ways of applying speculative fiction in health care research. Similar calls for greater methodological transparency have been made in related domains, including participatory eHealth design and human-computer interaction research [22,40,41]. *Second*, speculative fiction can function as a meaningful research tool only when its design is aligned with the study’s aims. Researchers need to ensure that the speculative fiction scenarios support the type of insights they want to generate. Depending on the goal of the study, for example, describing, comparing, and measuring effects, such techniques should be chosen deliberately during study preparation. *Third*, speculative fiction scenarios should be created or selected carefully, avoiding narratives that are unnecessarily extreme or disconnected from reality. A key consideration is the involvement of diverse voices in scenario development. Engaging stakeholders early helps ensure that scenarios feel grounded, balanced, and meaningful. Strong or exaggerated scenarios can be valuable tools for stimulating reflection but only when researchers are transparent about why such provocation is used. Speculative scenarios can influence or steer opinions, especially when they lean toward highly utopian or dystopian futures. Researchers must remain alert to this potential “dark side.”

### Limitations and Future Work

While this paper outlines insights learned from applying speculative fiction in health care research, these insights are primarily grounded in our own project experience. As such, several of the recommendations, such as the proposed grouping of speculative forms and the anticipated strengths and limitations presented in Table 1, reflect informed judgment based on practice rather than systematically gathered evidence. We therefore regard this paper as a starting point for a broader methodological conversation rather than a definitive framework. Additional perspectives from other research contexts, projects, and disciplines are essential to refine, challenge, and expand the ideas presented here. Continued sharing of practical experiences will strengthen the knowledge base and support more robust guidance for using speculative fiction in health care research.

Additionally, future research could compare how participants respond to different types of speculative scenarios. Such comparisons may help explain how speculative fiction influences reasoning, value expression, and sense-making. Although this was outside the scope of this paper, these studies would be valuable for assessing the method’s rigor and its ability to generate reliable insights about long-term attitudes, decision-making, or collaboration in health care.

Finally, cross-domain comparisons could deepen understanding of how speculative fiction works in health care compared with fields such as media studies, marketing, or public policy. Such studies may clarify how varying the level of utopian or dystopian elements shapes engagement and interpretation.

### Conclusions

This paper shows how speculative fiction can function as a structured yet imaginative tool for exploring uncertain futures in health care. By positioning speculative fiction within futures studies and participatory design traditions, and by outlining insights from a case study, the paper demonstrates how speculative scenarios can support reflection, surface values, and stimulate dialogue across diverse groups. Whether researchers choose to adapt existing scenarios or create new ones, speculative fiction can be tailored to different research aims, settings, and levels of analysis.

To advance this emerging field, a more systematic and transparent approach is needed. Developing guidelines would help ensure ethical clarity, balanced scenario design, and transparent reporting of researcher choices. Such guidance would not *replace* existing frameworks. Rather, it would *complement* them by offering a structured way to engage with uncertain futures and by strengthening the methodological grounding of speculative fiction in health care research. We therefore propose a 6-step reporting guide for using speculative fiction in health care research. With this paper, we contribute to the growing debate on future-oriented approaches in health care research and aim to strengthen the foundation for the thoughtful use of speculative fiction. We encourage other researchers to experiment with speculative fiction and to share their experiences with the health care research community to learn and advance its use.

## Abbreviations

AI: artificial intelligence
